# Diagnostic performance of lung ultrasound for systemic sclerosis-associated interstitial lung disease: an international cross-sectional study from two tertiary care centers

**DOI:** 10.1007/s10067-026-08191-y

**Published:** 2026-06-04

**Authors:** Davide Mohammad Reza Beigi, Jesús Loarce-Martos, Greta Pellegrino, Nicholas Landini, Valeria Panebianco, Carlos de la Puente Bujidos, Ana García de Vicente, Ilaria Bisconti, Francesca Romana Di Ciommo, Marius Cadar, Javier Arechavala Hita, Simona Truglia, Javier Bachiller-Corral, Fabrizio Conti, Valeria Riccieri

**Affiliations:** 1 Rheumatology Unit, Department of Medical and Cardiovascular Sciences, Sapienza University of Rome, Rome, Italy; 2 Rheumatology Clinic “Madonna Dello Scoglio”, Crotone, Italy; 3 Rheumatology Department, Hospital Universitario Ramón y Cajal, Madrid, Spain; 4 IRCCS Ospedale Galeazzi-Sant’Ambrogio, Milan, Italy; 5 Department of Biomedical and Clinical Sciences, University of Milan, Milan, Italy; 6 Department of Radiological, Oncological and Anatomopathological Sciences, Sapienza University of Rome, Rome, Italy; 7 Radiology Department, Hospital Universitario Ramón y Cajal, Madrid, Spain

**Keywords:** Diagnostic accuracy, High-resolution computed tomography, Interstitial lung disease, Lung ultrasound, Systemic sclerosis

## Abstract

**Objective:**

To assess the diagnostic accuracy of two lung ultrasound (LUS) scores (≥ 10 B-lines and pleural line abnormalities according to Fairchild's criteria) for detecting interstitial lung disease (ILD) in systemic sclerosis (SSc), when applied independently at two referral centers using a shared standardized protocol. Secondary aims included evaluating the diagnostic performance of their combined use and their association with ILD severity.

**Methods:**

This cross-sectional bi-center study included same-day LUS and high-resolution computed tomography (HRCT), using a 14-zone scanning protocol. ILD was defined as ≥ 10% lung involvement on HRCT. The diagnostic performance of each LUS score and their combination was assessed using *OR* and *AND* rules (the *OR* rule indicating positivity for either criterion, and the *AND* rule requiring both to be positive). Associations with disease severity, based on the Goh classification, were also explored.

**Results:**

ILD was present in 68% of the 108 patients included. The ≥ 10 B-lines cut-off showed 90.5% sensitivity and 84.4% specificity (AUC 0.875), while Fairchild's criteria achieved 93.2% sensitivity and 93.8% specificity (AUC 0.935). The OR rule yielded the highest sensitivity (95.9%) and NPV (89.7%), while the AND rule provided the highest specificity (96.9%) and PPV (98.5%). All patients with extensive ILD were positive for both scores.

**Conclusions:**

In this real-life multicenter setting, both LUS scores showed high and complementary diagnostic performance when applied within a standardized protocol. Their consistent association with ILD extent supports their clinical use as non-invasive tools for routine SSc-ILD assessment.

**Table:** 

**Key Points** • *Lung ultrasound shows high diagnostic accuracy for SSc-ILD across two international referral centres.* • *A standardized 14-zone protocol supports reproducible LUS performance.* • *≥ 10 B-lines and Fairchild’s criteria provide complementary diagnostic information.* • *Combining B-lines and pleural line criteria improves overall diagnostic performance.*

## Introduction

Systemic sclerosis (SSc) is a complex autoimmune connective tissue disease affecting several organs, with interstitial lung disease (ILD) representing one of the leading causes of morbidity and mortality [1, 2]. High-resolution computed tomography (HRCT) remains the gold standard for ILD diagnosis, staging and follow up, as well as for its prognostic role [3]; however, its repeated use is limited by ionizing radiation exposure, cost, and availability in longitudinal monitoring [4]. Pulmonary function tests (PFTs), including forced vital capacity (FVC) and diffusing capacity for carbon monoxide (DLCO), are widely used to monitor lung involvement, but may be insufficiently sensitive for early or subtle disease [5]. Moreover, FVC decline does not occur uniformly, and disease trajectories are often heterogeneous [6].

Lung ultrasound (LUS) has emerged as a noninvasive, radiation free, bedside imaging tool capable of detecting peripheral interstitial lung abnormalities in connective tissue diseases [7–9]. LUS can identify vertical artifacts known as B-lines, as well as pleural line (PL) alterations (PLA). B-lines are defined as discrete, laser-like vertical hyperechoic reverberation artifacts arising from the PL and extending to the bottom of the screen without fading [10]. Although their pathophysiological basis remains incompletely understood, recent experimental data suggest they reflect subpleural interstitial abnormalities and alterations in lung surface porosity, rather than thickened interlobular septa as previously assumed [11].

PL is normally visualized on LUS as a smooth, hyperechoic horizontal artifact, generated by the acoustic impedance difference between the visceral pleura and the aerated lung. PL abnormalities (PLA) include focal or diffused irregularity, thickening, coarseness and interruption of PL morphology [9]. However, their standardization and reproducibility have historically been more limited compared to B-lines. Some authors have reported a higher prevalence of PLA in fibrotic-predominant SSc-ILD, often in association with B-lines [12–14].

In a recent study, Rotondo et al. described a differential prevalence of LUS findings across HRCT patterns in SSc-ILD: B-lines were more frequently detected in NSIP (non-specific interstitial pneumonia) with ground glass opacities, whereas PLA (namely thickening and irregularity) were predominantly observed in UIP (usual interstitial pneumonia) and in patients fulfilling criteria for progressive fibrosing ILD. Importantly, both ultrasound findings were widely represented across radiological phenotypes and demonstrated a consistent ability to detect ILD, supporting their complementary role in the assessment of peripheral lung involvement [14].

However, to date, no consensus exists regarding the best LUS finding to use in SSc-ILD assessment and follow-up, whether B-lines, PLA, or both. Moreover, several LUS protocols have been proposed to evaluate ILD in SSc, but no international consensus currently exists regarding the optimal number of lung intercostal spaces (LIS) and the scanning technique to be employed [9]. Studies have used a wide range of scanning schemes, from limited 10-zone evaluations to extended protocols exploring up to 72 LIS [7]. This variability affects diagnostic reproducibility and complicates comparisons across studies. Increasing the number of scanned LIS generally improves sensitivity but also prolongs examination time, which may limit feasibility in routine settings [10]. Among the available scanning protocols, the simplified 14-LIS showed a good balance between sensitivity, specificity, and time consumption [15].

Regarding scoring systems, the presence of ≥ 10 B-lines on a 14-LIS protocol has been suggested as indicative of ILD, showing good concordance with significant abnormalities on HRCT (Warrick scores ≥ 7) and requiring about eight minutes of execution time [16].

On the other hand, Fairchild et al. developed diagnostic criteria for PLA evaluated across the same 14-LIS using a musculoskeletal preset, with an execution time of less than 15 min. Given their high specificity, these criteria may be particularly helpful in identifying fibrotic ILD even in patients with limited B-line involvement, complementing B-line–based scores [17]. These criteria were recently validated in a prospective study, confirming their diagnostic accuracy, reproducibility, and correlation with HRCT findings and DLCO in patients with SSc and idiopathic inflammatory myopathies, thus supporting their clinical applicability in SSc-ILD [18].

Our preliminary single-center analysis evaluated and compared the diagnostic performance of the two qualitative LUS scores (≥ 10 B-lines cut-off and Fairchild’s criteria) demonstrating good sensitivity for both and slightly higher specificity and accuracy for the PLA [19]. In a subsequent investigation, we focused on the correlation between quantitative LUS scores and automated quantitative chest tomography-defined ILD extent [20]. In this context, the present study was designed to assess the diagnostic accuracy of the two qualitative scoring systems in a larger, real-life multicenter cohort using a standardized 14-LIS protocol.

Based on these assumptions, our aim was to (1) reproduce and compare in a multicenter cohort the performance of Fairchild’s criteria and the ≥ 10 B-lines cut-off using a standardized 14-LIS protocol for ILD detection; (2) explore whether their combination improves diagnostic reliability, and (3) investigate the association of both LUS scores with ILD severity on HRCT, as well as with PFTs and clinical variables.

## Materials and methods

### Study design and population

This is a multicenter cross-sectional study in which data from consecutive adult patients with SSc (2013 ACR/EULAR criteria) [21] were collected. Each patient underwent both LUS and HRCT on the same day, with available PFTs performed within 1 month, at two tertiary rheumatology centers (Azienda Ospedaliero-Universitaria Policlinico Umberto I, Rome, Italy and Hospital Universitario Ramón y Cajal, Madrid, Spain) from January 2021 to December 2023. Exclusion criteria included lower respiratory tract infection within the past 6 months, history or echocardiographic signs of pulmonary arterial hypertension, other significant lung diseases unrelated to SSc (such as COPD or asthma), and a history of thoracic radiation therapy. Clinical, demographic, and laboratory data were collected at the time of LUS and HRCT.

### LUS examination

LUS was performed by experienced operators trained in ILD assessment. LUS was performed by two blinded operators at the Rome center (GP and DMRB), using a linear probe (12–15 MHz) mounted on a MyLab™Gamma ultrasound system (Esaote®, Geona, Italy). At the Madrid center, a single blinded operator (JLM) conducted the exam using a linear probe with a frequency range of 6–12 MHz on a GE Logiq™ E9 machine (GE HealthCare®, Chicago, Illinois, United States). A musculoskeletal preset was applied in both centers. Operators were blinded to clinical and HRCT data.

Image acquisition followed the simplified protocol described by Gutierrez et al., which includes the evaluation of 14-LIS: the 2nd parasternal, 4th mid-clavicular, 4th anterior and mid-axillary, and the 8th posterior axillary, subscapular, and paravertebral spaces on each hemithorax [15]. The probe was typically positioned in a sagittal orientation, resting between two ribs to span the intercostal space. When required by the patient's anatomy (e.g., limited acoustic windows), the probe was rotated to a transverse orientation parallel to the ribs to optimize PL visualization.

Two LUS-based criteria were applied for ILD detection. The first was the presence of ≥ 10 B-lines across the 14-LIS protocol. The second was the presence of PLA according to Fairchild’s criteria. At the time of data collection, the version employed corresponded to the pre-validation definition, as the formal validated criteria had not yet been published. The core features of the two versions are largely overlapping, and the subsequent prospective validation has confirmed the diagnostic value and applicability of the criteria used in this study [17, 18].

The diagnostic performance of a combined interpretation of the two LUS scores was also evaluated: the “OR rule” was defined as positivity for either B-lines ≥ 10 or Fairchild’s criteria, while the “AND rule” required both criteria to be present.

### HRCT assessment

HRCT scans were performed at each center according to local protocols, using multidetector scanners with thin-section acquisition (slice thickness ≤ 1.5 mm). Images were acquired at full inspiration with the patient in supine position. HRCT images were independently evaluated at each center by two thoracic radiologists, blinded to clinical and ultrasound data. Discrepancies were resolved by consensus. The presence and extent of ILD were assessed visually and quantified as the percentage of lung involvement, according to established criteria [22]. ILD was defined as present if fibrotic changes affected ≥ 10% of lung parenchyma. The ≥ 10% threshold was selected to capture clinically meaningful ILD and to reduce misclassification related to minimal or indeterminate abnormalities on HRCT. The severity of ILD was further classified using the Goh scoring system [23], which combines visual estimation of lung involvement on HRCT with FVC. According to this system, patients were stratified into limited (< 20% lung involvement) or extensive (≥ 20% lung involvement) disease. In cases where visual extent is indeterminate (20% lung involvement), FVC was used as an additional criterion to categorize disease severity.

## Statistical analysis

Statistical analyses were performed using GraphPad Prism 8.0. Continuous variables were expressed as median and interquartile range (IQR). Categorical variables were presented as frequencies and percentages. Sensitivity, specificity, positive predictive value (PPV), negative predictive value (NPV), accuracy, and area under the ROC curve (AUC) with corresponding 95% confidence interval (CI) were calculated for ≥ 10 B-lines, Fairchild’s criteria, and the combined OR and AND rules versus HRCT-confirmed ILD. For each LUS scoring system, 2× 2 contingency tables were constructed to derive raw classification counts, including true positives, false positives, true negatives, and false negatives. AUC values were interpreted according to conventional thresholds: 0.5–0.6 = poor, 0.6–0.7 = fair, 0.7–0.8 = acceptable, 0.8–0.9 = good, and > 0.9 = excellent [24]. Differences in diagnostic performance between the two LUS criteria were assessed by comparing sensitivity, specificity, predictive values, and overall discrimination on paired data using McNemar’s test. ROC curves were used to visually summarize the diagnostic performance of the evaluated LUS criteria, while sensitivity and specificity were considered the primary measures of accuracy. In patients with ILD, the relationship between LUS findings and disease extent (limited vs extensive, Goh classification) was evaluated by ROC analysis.

Univariate associations between LUS positivity and PFTs or clinical-demographic variables were assessed using chi-square/Fisher’s exact test or Mann–Whitney tests, as appropriate. Attempts at multivariable logistic regression were not feasible due to quasi-separation. The significance level was set at *p* < 0.05.

## Results

### Patient characteristics

A total of 108 SSc patients were included [81 (75%) in Rome, 27 (25%) in Madrid], 97 of whom were female (89%). Median age was 61 years (IQR 53–71), and median disease duration was 6 years (IQR 3–12). Seventy-four patients (68%) had ILD on HRCT, of whom 30 (40%) were classified as “extensive” according to the Goh score. A significantly higher prevalence of diffuse cutaneous SSc (47% vs. 15%, p 0.006) and a longer disease (8 vs. 3 years, p 0.012) and ILD duration (4 vs. 2 years, p 0.0295) were found in the Rome cohort, while patients from the Madrid cohort exhibited a significantly lower FVC% (median 86% vs. 96%, p 0.023). ILD prevalence and LUS score positivity rates were comparable between centers. Characteristics of the study population overall and by center are shown in Table 1.

**Table 1:** Characteristics of study population

Characteristic	Overall (*n* = 108)	Rome (*n* = 81)	Madrid (*n* = 27)	*p*-value
Female/Male, No. (%)	12 (11)/96 (89)	10 (12)/71 (88)	2 (7)/25 (93)	0.7237
Age, years, median (IQR)	61 (53–70)	63 (53–72)	57 (54–66)	0.322
Disease duration, years, median (IQR)	6 (3–12)	8 (4–14)	3 (1–9)	0.0119
Diffuse cutaneous form, No. (%)	42 (39)	38 (47)	4 (15)	0.0062
Smoking habit (current or previous), No. (%)	32 (30)	26 (32)	6 (22)	0.4654
Anti-Scl-70 antibodies positivity, No. (%)	40 (37)	33 (41)	7 (26)	0.25
Anti-centromere antibodies positivity, No. (%)	34 (31)	23 (28)	11 (41)	0.3386
ILD presence at CT, No. (%)	74 (68)	56 (69)	18 (67)	1
ILD duration, years, median (IQR)	3 (1–7)	4 (1–8)	2 (0–4)	0.0295
Extensive disease according to Goh score, No. (%)	30 (40)	22 (39)	8 (44)	0.8275
Digital ulcers, No. (%)	30 (28)	23 (28)	7 (26)	1
Immunosuppressant therapy, No. (%)	59 (55)	45 (56)	14 (52)	0.9111
Glucocorticoid therapy, No. (%)	37 (34)	28 (35)	9 (33)	1
FVC %, predicted, median (IQR)	94 (77–108)	96 (77–110)	86 (75–94)	0.0227
DLCO %, predicted, median (IQR)	72 (58–84)	72 (58–84)	75 (60–85)	0.8
Fairchild’s criteria positivity, No. (%)	72 (67)	53 (65)	19 (70)	0.8749
B-lines ≥ 10, No. (%)	72 (67)	56 (69)	16 (59)	0.4287

*ILD* Interstitial lung disease, *CT* Chest tomography, *FVC* Forced vital capacity, *DLCO* Diffusing capacity for CO

### LUS findings

Fairchild’s criteria and ≥ 10 B-lines cut-off were positive in 72 (66%) patients in the whole population (not the same patients). In Rome 56/81 (70%) patients were positive for B-lines and 53/81 (66%) for Fairchild’s criteria, while 16/27 (59%) and 19/27 (70%) were positive for the respective scores in Madrid.

### Diagnostic performance of LUS scores

The ≥ 10 B-lines cut-off showed sensitivity 90.5%, specificity 84.4%, PPV 93.1%, NPV 79.4%, accuracy 88.7%, and AUC 0.875. This corresponded to 67 true positives (TP), 5 false positives (FP), 27 true negatives (TN), and 7 false negatives (FN).

Fairchild’s criteria had sensitivity 93.2%, specificity 93.8%, PPV 97.2%, NPV 85.7%, accuracy 93.4%, and AUC 0.935, with a classification of 69 TP, 2 FP, 30 TN, and 5 FN (Table 2).

**Table 2:** Diagnostic performance of combined LUS rules versus CT-confirmed ILD (95% confidence interval)

Rule	Sensitivity	Specificity	PPV	NPV	Accuracy	AUC
≥ 10 B-lines	0.905 (0.817–0.953)	0.844 (0.682–0.931)	0.931 (0.848–0.970)	0.794 (0.632–0.897)	0.887 (0.812–0.934)	0.875
Fairchild’s criteria	0.932 (0.851–0.971)	0.938 (0.799–0.983)	0.972 (0.903–0.992)	0.857 (0.706–0.937)	0.934 (0.870–0.968)	0.935
OR	0.959 (0.887–0.986)	0.812 (0.647–0.911)	0.922 (0.840–0.964)	0.897 (0.736–0.964)	0.915 (0.846–0.955)	0.886
AND	0.878 (0.785–0.935)	0.969 (0.843–0.994)	0.985 (0.919–0.997)	0.775 (0.625–0.877)	0.906 (0.835–0.948)	0.924

*LUS* Lung ultrasound, *CT* Chest tomography, *ILD* Interstitial lung disease, *PPV* Positive predictive value, *NPV* Negative predictive value, *AUC* Area under the curve, *OR* either ≥ B-lines cut-off or Fairchild’s criteria are present, *AND* both ≥ B-lines cut-off and Fairchild’s criteria are present

The comparison of the two ROC curves showed no statistically significant difference between the AUCs of the ≥ 10 B-lines cut-off and Fairchild’s criteria (p 0.79).

When combining the two LUS scoring systems, diagnostic performance was assessed using both “OR” and “AND” rules. Applying the “OR” rule (defined as the presence of either ≥ 10 B-lines or Fairchild’s criteria positivity) resulted in a sensitivity of 95.9%, specificity of 81.3%, PPV of 92.2%, NPV of 89.7%, accuracy of 91.5%, and AUC of 0.886. This rule correctly identified 71 TP and 26 TN, with 6 FP and 3 FN.

**Fig. 1:**
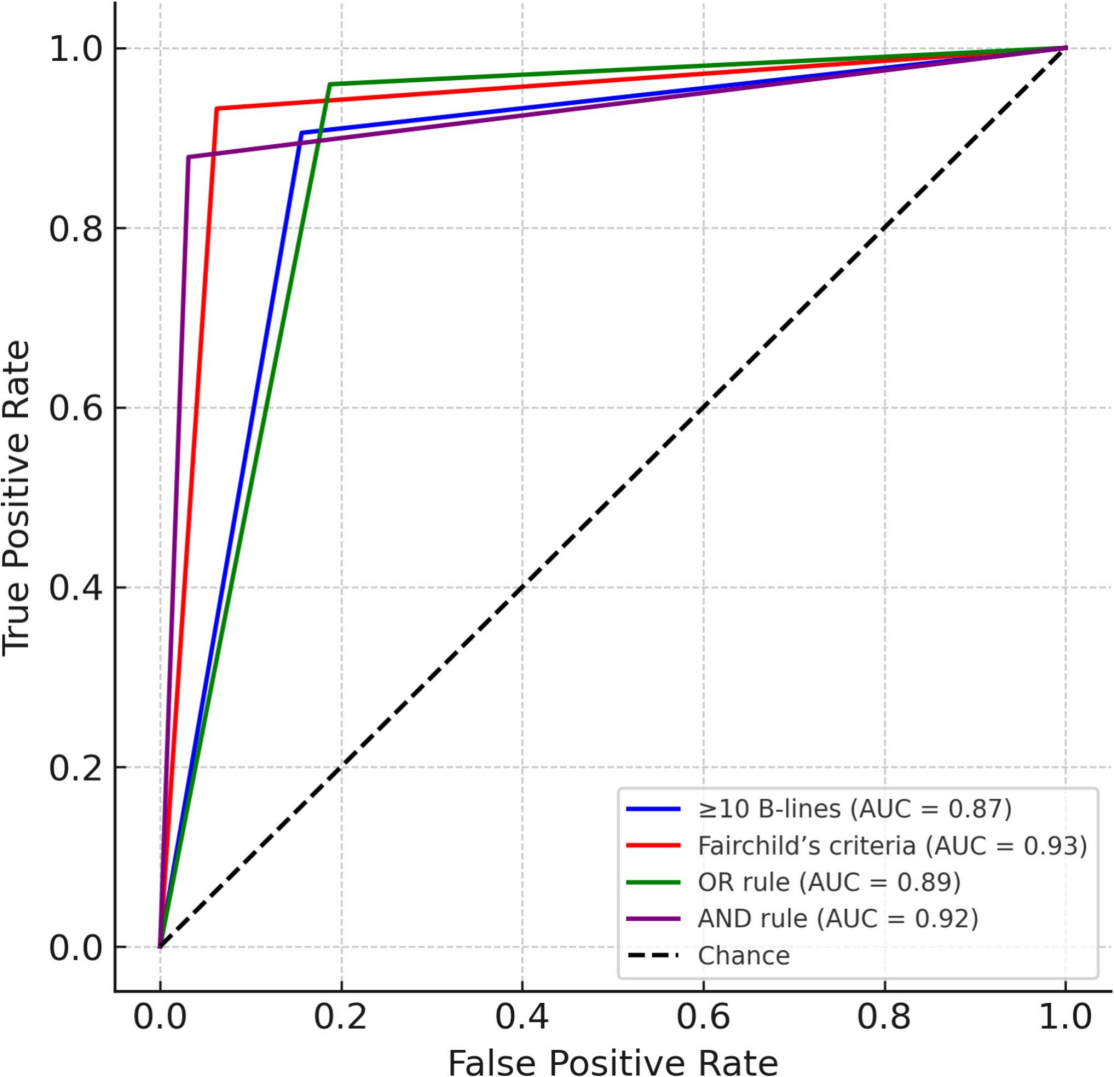
ROC curves comparing single and combined LUS scores (≥ 10 B-lines, Fairchild’s criteria, OR/AND rules) versus HRCT-defined ILD. ROC, receiver operating characteristic; LUS, lung ultrasound; HRCT, high resolution chest tomography; ILD, interstitial lung disease

Conversely, the “AND” rule (requiring both criteria to be positive) yielded a sensitivity of 87.8% and a markedly higher specificity of 96.9%, with a PPV of 98.5%, NPV of 77.5%, accuracy of 90.6%, and AUC of 0.924. This approach resulted in 65 TP, 1 FP, 31 TN, and 9 FN (Table 2). Figure 1 reports the ROC curves of all LUS scores.

### Association with extensive disease, PFTs and clinical variables

All 30 patients (100%) classified as having extensive ILD according to the Goh score were positive for both the ≥ 10 B-lines cut-off and Fairchild’s PL criteria. To further assess the association between LUS criteria and ILD severity, their ability to discriminate between limited and extensive ILD in the whole cohort was evaluated. The AUC was 0.727 (95% CI 0.61—0.84, p 0.002) for both the ≥ 10 B-lines cut-off and Fairchild’s criteria, 0.688 (0.56—0.81, p 0.006) for the OR rule, and 0.766 (0.65—0.88, *p* < 0.001) for AND rule.

Patients with ≥ 10 B-lines had lower median predicted %FVC [90 (71—103.5) vs. 99 (89—114.5, *p* < 0.0037) and %DLCO [68 (52.25—82) vs. 79 (63—91), *p* < 0.0092)] compared to those with < 10 B-lines. Similarly, patients positive for Fairchild’s criteria had lower %FVC [88 (71—99) vs. 101 (91—114.5), p 0.00018) and %DLCO [68 (52.25—75) vs 82 (63—93), p 0.00168] compared to those who did not meet the criteria. Additionally, anti-Scl-70 positivity, digital ulcers history, crackles and immunosuppressant assumption were more frequent in LUS-positive subjects (Tables 3 and 4).

**Table 3:** Significant differences in clinical variables according to ≥ 10 B-lines presence

Variable	≥ 10 B-lines (*N* = 72)	< 10 B-lines (*N* = 36)	*p*-value
Anti-Scl-70 positivity, No. (%)	36/72 (50%)	4/36 (11%)	0.0002
Auscultatory crackles, No. (%)	56/72 (78%)	14/36 (39%)	0.0002
Digital ulcers history, No. (%)	26/72 (36%)	4/36 (11%)	0.0122
Ongoing immunosuppressive therapy, No. (%)	49/72 (68%)	10/36 (28%)	0.0002
Ongoing glucocorticoid therapy, No. (%)	30/72 (42%)	7/36 (19%)	0.0376

**Table 4:** Significant differences in clinical variables according to Fairchild’s criteria positivity

Variable	Fairchild’s-positive (*N* = 72)	Fairchild’s-negative (*N* = 36)	*p*-value
Anti-Scl-70 positivity, No. (%)	34/72 (47%)	6/36 (17%)	0.0039
Auscultatory crackles, No. (%)	57/72 (79%)	13/36 (36%)	< 0.0001
Digital ulcers history, No. (%)	26/72 (36%)	4/36 (11%)	0.0122
Ongoing immunosuppressive therapy, No. (%)	50/72 (69%)	9/36 (25%)	< 0.0001

## Discussion

This multicenter, real-life cross-sectional study assessed the diagnostic performance of two qualitative LUS criteria for detecting SSc-ILD using a shared 14-zone scanning protocol. Both the ≥ 10 B-lines cut-off and Fairchild’s PL criteria showed good accuracy when compared to HRCT-defined ILD.

The ≥ 10 B-lines threshold yielded a sensitivity of 90.5% and a specificity of 84.4% for ILD detection, with an AUC of 0.875. Previously, employing the same scanning protocol, Tardella et al. found that ≥ 10 B-lines were predictive of radiological evidence of ILD, defined as a Warrick score ≥ 7 [16]. In the context of B-lines assessment, Gargani et al. have recently highlighted the pivotal role of protocol standardization to enhance methodological consistency and enable meaningful comparisons across studies: while a 58-LIS protocol achieved very high sensitivity (~ 97%) for ILD detection using a ≥ 10 B-lines threshold, it did so at the cost of reduced specificity (~ 64%). In contrast, applying a simplified 14-LIS approach with a ≥ 6 B-lines threshold improved specificity to ~ 85%, while maintaining sensitivity at ~ 90% [25]. Findings from this study, based on a ≥ 10 B-lines threshold within a 14-LIS protocol, offer a comparable diagnostic yield, highlighting that simplified protocols can maintain high diagnostic performance while improving feasibility in clinical practice.

Fairchild’s criteria also showed high diagnostic accuracy, with 93.2% sensitivity, 93.8% specificity, and an AUC of 0.935, which was slightly higher than that of the B-lines cut-off, although the difference was not statistically significant. These results are consistent with those reported by Fairchild et al. and align with our previous single-center experience, confirming the relevance of PLA as reliable LUS markers of SSc-ILD [17]. Shortly after this study, the LUS-ILD-24 criteria—a prospectively validated refinement of the original scoring system—were tested in a single-center cohort of patients with SSc and idiopathic inflammatory myopathies. While sensitivity remained high (92.4–95.5%), specificity was slightly reduced (82.8–86.2%) compared to the reference report [18]. Despite this, the criteria confirmed good diagnostic performance and reproducibility, supporting the robustness of the original version adopted in this study and its clinical applicability within standardized LUS protocols. Recent work by Rotondo et al. described differences in the prevalence of PLA across HRCT patterns in SSc-ILD, with a higher representation in fibrotic-predominant disease; however, PLA were observed across the spectrum of SSc-ILD and showed a consistent ability to detect ILD, supporting their complementary role alongside B-lines in the assessment of peripheral lung involvement [14].

When combining both LUS scores, a complementary diagnostic profile was observed. The OR combination yielded the highest sensitivity (95.9%) and NPV (89.7%), whereas AND combination resulted in the highest specificity (96.9%) and PPV (98.5%). Although the AND rule yielded a numerically higher AUC (0.924 vs. 0.886 for the OR rule), the difference did not reach statistical significance. This flexible diagnostic strategy allows clinicians to adapt LUS interpretation based on clinical priorities, favoring the OR rule in screening or high-sensitivity settings, and the AND rule in confirmatory contexts or when HRCT is not readily available. To our knowledge, this is the first study to formally compare these two qualitative LUS scores in combination, supporting a tailored, protocol-based application in clinically indicated SSc-ILD assessment, while prospective longitudinal studies are needed before fixed LUS monitoring intervals can be recommended.

Both LUS scores showed strong concordance with ILD severity, as defined by the Goh visual classification on HRCT. All patients classified as having extensive disease were positive for both the ≥ 10 B-lines cut-off and Fairchild’s criteria. In line with the AUC values observed for detecting extensive ILD, these findings support the potential role of LUS both in screening for ILD and in identifying patients who are more likely to exhibit extensive pulmonary involvement on HRCT. In addition, both LUS scores were significantly associated with reduced pulmonary function parameters, including lower FVC and DLCO, as well as with other clinical features typically related to fibrotic or more active disease. Anti-Scl-70 antibody positivity, digital ulcers, auscultatory crackles, and ongoing immunosuppressive treatment were all more frequent among LUS-positive patients. These findings further support the role of LUS in capturing clinically relevant disease phenotypes in SSc-ILD.

### Strengths and limitations

The strengths of this study include its multicenter design, the simultaneous evaluation of two complementary LUS criteria, and the use of a shared, evidence-based 14-zone scanning protocol. All ultrasound exams were performed by operators trained in ILD assessment, and HRCT was conducted on the same day as LUS, ensuring optimal temporal alignment. Notably, the consistency of findings across two independent centers, each applying the same LUS protocol in a real-life setting, supports the applicability and robustness of these scores across different clinical environments. To our knowledge, this is the first study to systematically apply both the ≥ 10 B-lines cut-off and Fairchild’s PL criteria—each previously validated independently—within a standardized, pragmatic framework across different centers. In a field still marked by methodological heterogeneity, where most studies assess only B-lines and use variable scanning schemes, these findings offer a first real-world demonstration of feasible LUS standardization for SSc-ILD assessment.

Nevertheless, several limitations should be acknowledged. First, although all patients underwent both HRCT and LUS on the same day following a systematic protocol in both centers, imaging was clinically indicated either for baseline ILD screening or for follow-up evaluation. Despite the efforts to reduce bias by indication, the study population may represent a higher-risk subgroup, resulting in an elevated ILD prevalence (~ 70%) and limiting generalizability to unselected SSc populations. In addition, the relevant proportion of patients with extensive ILD may have increased the apparent diagnostic performance of LUS, as more advanced lung involvement is more likely to generate peripheral and subpleural abnormalities detectable by ultrasound. This case-mix is expected to inflate PPV and may yield different NPV estimates than those observed in unselected screening populations. Therefore, the performance metrics should be interpreted within a clinically indicated setting, where LUS is most often applied in routine care. Nonetheless, this design closely mirrors the setting of recent validation of Fairchild’s criteria, which also targeted individuals at risk for or with known ILD [18]. Therefore, the applicability and reproducibility of our findings remain robust within clinically relevant scenarios.

Second, although both sites applied the same 14-LIS protocol, minor differences in ultrasound equipment (including probe frequency and preset) may have influenced image acquisition and interpretation; however, the exact comparability of machine settings across centers could not be fully verified and remains a source of potential heterogeneity. Nevertheless, in a real-life setting, different scanners may be available in different centers: hence, testing the performance of LUS with different devices may support the generalizability of the results. Third, LUS was performed by two blinded operators in Rome and by a single operator in Madrid; although all were experienced and trained in ILD assessment, inter-operator variability was not formally assessed.

Furthermore, the available sample was determined by the number of consecutive eligible patients evaluated during the study period, rather than by a formal a priori power calculation. Accordingly, the study was intended primarily to estimate the diagnostic performance of the prespecified LUS criteria against HRCT-defined ILD, not to provide a powered comparison between the two LUS scores. The contribution of the two centers was also unbalanced, with Rome accounting for most patients. While LUS and HRCT were performed on the same day in all cases, enhancing temporal validity, the generalizability of these findings to prospective or broader clinical settings remains to be demonstrated. Finally, although some baseline differences were observed between centers, including a higher prevalence of diffuse cutaneous disease and longer disease and ILD duration in Rome, and lower FVC% values in Madrid, ILD prevalence and LUS positivity rates were comparable. This supports the robustness of LUS scoring systems across heterogeneous real-life cohorts, although prospective validation in more balanced populations is warranted.

## Conclusion

In conclusion, both the ≥ 10 B-lines and Fairchild’s PL criteria proved to be accurate and complementary LUS tools for SSc-ILD detection. Applied within a standardized 14-LIS protocol, they offer a feasible and adaptable approach for clinical use. Their association with ILD severity and clinical features further reinforces the role of LUS as a non-invasive, bedside tool for disease assessment in SSc-ILD. These findings, derived from a real-world multicenter cohort, support the potential implementation of standardized LUS protocols in routine rheumatologic practice. Future prospective studies are warranted to confirm these results and evaluate their prognostic and longitudinal utility.

## Data Availability

The datasets generated and/or analyzed during the current study are available from the corresponding author on reasonable request.
